# Toward Livestock Supply Chain Sustainability: A Case Study on Supply Chain Coordination and Sustainable Development in the Pig Sector in China

**DOI:** 10.3390/ijerph16183241

**Published:** 2019-09-04

**Authors:** Ni Zhuo, Chen Ji

**Affiliations:** 1 Department of Agricultural Economics and Management, School of Public Administration, Zhejiang University, Hangzhou 310058, China; 2 China Academy for Rural Development (CARD), Zhejiang University, Hangzhou 310058, China

**Keywords:** supply chain coordination, supply chain sustainability, livestock industry

## Abstract

Stricter environmental regulations on livestock production pollution have changed the sustainable practices of livestock supply chain stakeholders. By adopting three cases in China’s livestock supply chain, this study explores how supply chain coordination facilitates sustainable development of livestock production in China. It is found that close supply chain coordination and the capabilities of the core companies jointly contribute to supply chain sustainability. Thus, this research has theoretical significance in explaining the roles of supply chain coordination and core company capabilities in driving supply chain sustainability, which is not completely understood thus far. This study also has practical implications for livestock supply chain stakeholders and the government in terms of improving supply chain sustainability via closer supply chain coordination and enhancing the capabilities of the core companies involved.

## 1. Introduction

In recent years, the Chinese government has enacted and implemented a series of environmental regulations to improve the country’s environmental conditions. In October 2013, the state council issued a regulation on environmental pollution produced by the livestock sector (hereafter called the “Regulation”). This is considered the strictest environmental control regulation in the livestock sector. Therefore, there is an emerging need for supply chain members to cope with the Regulation to achieve sustainable development in the livestock sector.

A sustainable supply chain performance refers to traditional measures of profit and loss, as well as an expanded conceptualization of performance that includes social and natural dimensions [1]. It is commonly agreed that the sustainable outcome of a supply chain should include sustainable economic performance [2], social performance [3], and environmental performance [4]. This is known as the triple bottom line in supply chain sustainability literature.

Current studies have discussed the factors that may drive sustainability such as climatic change [5], uncertainties [6], institutional changes [7], governance structures [8], and transparency in the food supply chain [9]. However, discussion on potential contributions from supply chain coordination remains limited. Supply chain coordination is a theme that receives wide attention in supply chain management studies, and its outcome includes relationship satisfaction [10], food safety [11], and enhancement of supply chain performance [12]. Supply chain sustainability as an outcome of supply chain coordination has not captured sufficient attention [13]. Through a literature review, it was found that supply chain members may work together to obtain capabilities at the supply chain level, and the capabilities may contribute to the sustainable performance of the supply chain [3].

Therefore, by adopting three cases in the pork supply chain in China, this study first tries to understand how supply chain members in the pig sector work together to build a more sustainable pork supply chain. Then, the study explores the relationship between supply chain coordination and supply chain sustainability by providing empirical evidence from the pig sector in China. At the same time, this study tries to reveal the underlying mechanism using dynamic capabilities theory.

## 2. Literature Review

### 2.1. Agrifood Supply Chain Sustainability

An increasing population, deficiency of natural resources, and climate change require organizations in the agrifood sector to redesign their current supply chains to involve social, economic, and environmental perspectives in their performance [14]. Crowder and Reganold pointed out that sustainability is particularly relevant for the agrifood industry because sustainability contributes to agrifood companies in gaining competitive advantages in the market [15]. This is thought to be closely associated with quality improvement. Firms that adopt self-regulation in sustainability practices are able to better anticipate regulation changes from the government [16]. Thus, these firms can voluntarily improve traceability and accountability across the supply chain, and ensure consistent quality and a reliable supply [17].

However, the issues of supply chain sustainability in agrifood industries have not been adequately empirically studied [18]. Current studies have primarily attempted to understand the environmental sustainability practices in developed countries using life cycle analysis (LCA) methods [19], while empirical evidence is lacking in emerging economies [20]. From the literature review, it was found that on the one hand, scholars have indicated that environmental practices should be performed by different stakeholders of the supply chain so that sustainability of the agrifood chain will be achieved [21]. On the other hand, scholars have agreed on some dimensions to evaluate the sustainable performance of supply chains. For example, environmental sustainability is usually measured by on-farm and off-farm environmental protection practices [22], lower emissions of pollutants [23], adoption of organic fertilizer [24], use of third-party certification [25], and other instruments [26]. The social dimension of sustainable development involves human rights [25], an increase in social welfare [27], the development of local communities [28], and customer health and safety [29]. Economic performance includes productivity, financial outcomes, and customer satisfaction [30].

### 2.2. Agrifood Supply Chain Coordination

Supply chain coordination is not a new concept in supply chain management. It refers to a collection of formal or informal institutional relationships among supply chain members [31]. The coordinated supply chain relationships in the food sector that have been most widely reported in the literature include market relations, short-term contracts, long-term contracts, joint ventures, strategic alliances, and vertical integration [32]. There are several outcomes of supply chain coordination mentioned in the literature, including the relationships between supply chain coordination and supply chain performance [33], supply chain coordination and supply chain relationship satisfaction [34], and supply chain coordination and food safety [11].

The relationship between supply chain coordination and sustainability has been increasingly discussed in the recent literature. Evidence has shown that business-to-business collaboration in the food industries leads to improvements in economic, environmental, and social standards through effective communication between farmers and traders [35]. It is proposed that the interplay of collaboration behaviors will influence agrifood supply chain sustainability, while empirical evidence is not provided [13]. The mechanism of collaboration will influence supply chain sustainability and needs more investigation. Theories such as the dependency theory and transaction cost economy theories can be adopted [36].

### 2.3. Dynamic Capabilities of Organizations

Helfat and Winter defined dynamic capacity as the capacity of an organization to purposefully create, extend, and modify its resource base [37]. Dynamic capability theory is rooted in the resource-based view (RBV) [38], which posits that firms combine bundles of valuable, rare, inimitable, and non-substitutable resources in an effort to gain or maintain a competitive advantage [39]. Tsai et al. stated that dynamic capability is a factor that influences organizational sustainability [40]. Dynamic capability theory tackles this challenge by reasoning that organizations consistently operating in dynamic environments create and recombine their resources in new ways [2].

In the agrifood supply chain, focal companies usually drive the main changes in the supply chain, such as supply chain integration and food safety certification adoption. These companies were asked to consider the environmental and social problems present in their entire supply chain [41].

### 2.4. Relationships Among Supply Chain Coordination, Dynamic Capabilities of Organization, and Supply Chain Sustainability

Supply chain members jointly learn how to build capabilities through learning loops for innovations that will help the entire supply chain become more sustainable [13]. Focal companies are supply chain members that provide leadership for the supply chain, specify supply chain policies to other members, and exercise control over the supply chain’s decisions and activities [42].

Soylu et al. pointed out that supply chain collaboration is a common way for companies throughout the supply chain to share information, make strategic alliances, and reduce overall costs, as well as to improve supply chain sustainability [43]. Azevedo et al. pointed out that collaboration is seen as necessary to develop, apply, and establish new innovative ideas and practices, and is linked to the socioeconomic and ecological sustainability of the agrifood system [44]. Chen et al. stated that one of the reasons that companies perform supply chain collaboration is to achieve sustainability [13]. This includes collaboration with suppliers, customers, competitors, and other organizations. This will enrich the companies’ resources and enhance their capability for achieving an improved sustainable performance.

Therefore, the research question of this study is:

RQ: How is supply chain sustainability jointly achieved through building the supply chain coordination and dynamic capabilities of the core companies?

## 3. Data and Methodology

### 3.1. Case Study Method

According to Yin [45], a case study design should be considered when (a) the focus of the study is to answer “how” and “why” questions, (b) you cannot manipulate the behavior of those involved in the study, (c) you want to cover contextual conditions because you believe they are relevant to the phenomenon under study, or (d) the boundaries are not clear between the phenomenon and context. A multiple-case study enables the researcher to explore differences within and between cases. The goal is to replicate findings across cases. The researcher can predict similar results across cases or predict contrasting results based on a theory [45]. Therefore, a multiple-case method is appropriate to be adopted in this study because there is a “how” research question that requires exploration of the mechanism of livestock supply chain sustainability.

Three different supply chain modes in the pig sector in China were selected for study: (1) “Vertical integration” mode (Mu’yuan Food Co., Ltd.), (2) “company + local family-owned commercial farms” mode (Wen’shi Food Co., Ltd. (Huai’an)), and (3) “cooperative + farmers + company” mode (Chun’ran Agri-Food Tech Co., Ltd.). Table 1 provides the basic information of the three cases. These three cases all undertake supply chain coordination to achieve sustainable development. To do this research, we obtained firsthand data from field studies and secondhand data from the official websites of the companies.

### 3.2. Data Collection

We conducted 15 interviews (four interviewees from Mu’yuan, six interviewees from Wen’shi (Huai’an), and five interviewees from Chun’ran) in total from May 2018 to April 2019. To ensure a comprehensive view was captured, the interviewees included all managers of the three companies, the presidents of cooperatives, some core members of the local family-owned commercial farms, and some farmers. All of them were interviewed twice or more.

Research instruments included face-to-face semi-structured interviews lasting 60–120 mins per interview and archival data from the official websites of the companies or from direct observation. Our interview protocol addressed the following key issues: (1) The companies’ profiles, (2) their supply chain coordination, and (3) their sustainable development.

We carried out at least two rounds of data collection/field visits for each company (Table 1). Generally, in the first round of data collection, we tried to understand the supply chain modes of the case companies. In the second round of field visits, we collected data with regard to supply chain coordination and sustainable development. The field researchers (coauthors) visited each of the three companies. For each visit, the field researchers recorded field notes based on direct observations of the case company’s operations.

### 3.3. Data Analysis

The study adopted case analysis and cross-case analysis to analyze the data. It carried out within-case analysis first for each of the three companies. Each field researcher individually coded the data, and then the coded data between the coauthors were compared to ensure consistency and inter-coder reliability. Disagreements were resolved along the way.

Within-case analysis gained a broad understanding of the business models of each case company and the supply chain coordination and sustainable development of the cases. Cross-case analysis was performed and the findings tabulated to identify the mechanism of how companies in the pork supply chain in China achieve sustainable development with supply chain coordination.

## 4. Descriptions of Three Case Companies

### 4.1. Mu’yuan Co., Ltd.

Mu’yuan Co., Ltd., was founded in 1992. It is a top-listed agricultural company in China. Its businesses include feed production, pig production, and pig slaughtering. The feed production volume of Mu’yuan reaches 5 million tonnes annually, and pig production reaches 10 million heads annually. The number of pigs slaughtered reaches 1 million heads. The supply chain of Mu’yuan is integrated: The company manages the feed production, pig production, and slaughtering itself. There are branches of Mu’yuan across nine provinces in the country. Thus, Mu’yuan effectively handles pig production safety. Mu’yuan puts emphasis on the recycling economy. The company has developed a way to use production wastes as fertilizers for planting, thus driving the surrounding farmers to vigorously develop an ecological agriculture.

### 4.2. Wen’shi Co., Ltd.

Wen’shi Food Co., Ltd., started pig production in Jiangsu Province in 2006. Relying on its “company + local family-owned + commercial farms” mode, Wen’shi provides standardized pigs to local family-owned commercial farms, and shares profits with them so that farmers can ensure the safety and quality of pig production. Thus, Wen’shi has successfully rooted this innovative mode into the Southeast area. Taking Jiangsu province as an example, there are eight regional subcompanies in the province with more than 3000 staff and nearly 200 local family-owned commercial farms. Wen’shi puts great effort into practicing sustainable pig production, and it leads the local farms in changing traditional ways of pig farming.

### 4.3. Chun’ran Agri-Food Tech Co., Ltd.

Long’zhu cooperative (hereafter Long’zhu) was founded in 2010 with a group of 36 farmer members in Qu’zhou city, Zhejiang province. The initial purpose of establishing the cooperative was to purchase feed together from Ke’sheng, which is a local feed company. With the development of Long’zhu, the president (Mr. Zhao Chungen, hereafter Zhao) found that market price fluctuations made the profits of farmers uncertain, so he decided to create downstream integration to generate a profit premium for farmer members. Zhao started Chun’ran Agro-Tech Co., Ltd., and registered the pork brand Jiu’hao’mu’chang to sell premium quality pork to the marketplace. (Long’zhu collaborated with the Animal Science School of Zhejiang University to create a type of feed that includes tea leaves, which makes the pork taste better; Zhao made the pork a premium product in the marketplace.) Zhao also started a pig production waste company (called Kai’qi Energy Co., Ltd.) to help member farmers avoid risks from the changing environmental policies of the government.

## 5. Cross-Case Analyses

### 5.1. Supply Chain Coordination of Case Companies

In this section, the supply chain coordination of the three companies is described (see Table 2).

#### 5.1.1. Supply Chain Coordination of Mu’yuan

Mu’yuan is a traditional pig production company, but it integrates both upstream and downstream stakeholders. It has a very stable relationship with producers because farmers produce pigs as required by Mu’yuan, and they abide by all safety standards dictated by Mu’yuan. In fact, the upstream farmers are “workers” for Mu’yuan, they do not actually own their farms or produce pigs for themselves. Mu’yuan employs the farmers to produce pigs for them on farms owned by Mu’yuan, and the farmers’ income is not associated with the number of pigs sold.

#### 5.1.2. Supply Chain Coordination of Wen’shi

Wen’shi has a close relationship with local family-owned commercial farms. To be more specific, Wen’shi chooses to partner with local family-owned commercial farms, which produce between 1000 to 170,000 heads annually. Wen’shi and its partnering farms sign contracts to share profits and share risk. Contracted local farms receive breed, feed, and other inputs from Wen’shi for free, and they establish production farms according to the instructions of Wen’shi. Wen’shi monitors all production processes of its contracted farms in order to ensure food safety.

Wen’shi shields its farmers from market risks, and it pays for the farmers to fatten pigs over 155 days based on three categories of quality: I, II, and III. The price tiers are 14 RMB/kg for quality class I, 12 RMB/kg for quality class II, and price to be determined for quality class III. In this way, Wen’shi encourages the farmers to produce pigs of high quality. Wen’shi ensures that the farmers receive profits by adjusting the purchasing price to the market price. The costs of breaching a contract for farmers and for Wen’shi are both high. If farmers breach a contract, they will need to pay all production costs plus 20% of the value of a class-I pig. If Wen’shi breaches a contract, it pays the farmers at least US $335 plus a value adjustment for the market.

#### 5.1.3. Supply Chain Coordination of Chun’ran

Chun’ran manages the supply chain coordination with farms through the Long’zhu cooperative. There are 55 cooperative member farmers in the Long’zhu cooperative, and the primary link between the cooperative and Chun’ran is the collaborative purchase of feed products in order to achieve economies of scale. Mr. Zhao Chungen aims to sell premium pork to the market, and thus 10% of the cooperative farms have a market relationship with Chun’ran.

### 5.2. Dynamic Capabilities of Organizations

In this section, the dynamic capabilities of the three companies is described (see Table 3).

#### 5.2.1. Capability of Disposing Wastes of Three Companies

Regarding the capability of disposing of production wastes, Mu’yuan enjoys natural advantages because the lands in Henan province are almost plain and the weather is suitable to producing crops. The Research and Development group of Mu’yuan converts the production wastes into organic fertilizer, which is used for crop production, and methane, which is used for some farm management utilities. Thanks to the dry weather in Henan province, the organic fertilizer is not easily washed away.

Wen’shi pays attention to the treatment of liquid wastes, gas wastes, and residue wastes. The solution to liquid wastes is to use an urban sewage treatment system that can separate open ditches and underground pipes so that rainwater does not mix with liquid waste. The solution to gas waste is to use a centralized liquid spray system to cover the gas waste so that the transmission distance of gas waste is largely reduced. The waste residue is directly returned to the field by using a vertical fermentation tank for fermentation treatment.

Chun’ran invested US $2.9 million to start a pig production waste recycling company (Kai’qi Energy), which helps cooperative member farmers to dispose of pig production waste. All member farmers transport their production waste to Kai’qi Energy Co., Ltd. This disposal service is free, so member farmers need not worry about the cost of waste disposal. In addition, the energy produced after the waste process can be used by the farmers because Chun’ran inputs the electricity of Kai’qi Energy Co., Ltd., into the national electricity network.

*“We feel proud of our recycling system, which received awards from local government and was set as an example in livestock waste recycling. With this plant, our member farms were successfully shielded from stricter environmental policy risks, and our cooperative became even more prosperous,”* said Zhao.

#### 5.2.2. Capability of Establishing Sustainable Production Base

Mu’yuan has created its own production environment management system, which consists of three sections. The first is the ventilation system. A siphoning system in the production house helps with air circulation, and the air inlet window together with the exhaust window strengthens the ventilation. The second section is a device to ensure humidity, including wire rope and a wet sack on the wire rope. These help to add humidity to the pig house. The third section is a pig house heating and insulation device, which includes a main outlet pipe and main return pipe. A water heater is connected to the main outlet pipe and main return pipe. The main outlet pipe is connected to multiple thin heat pipes, the heat pipes are laid parallel to each other on the back of the pig net bed, and the heat is cooled. The other end of the tube is connected to the main return pipe.

Wen’shi has built its own production house. The pig house is designed with a diffused ventilation system and is kept at a constant temperature of 25 °C. It is equipped with a leaking plate. The urine and feces can be leaked through the plates. A urinary septic tank is placed under the plates. The feces is softened by the urine and then transferred to a fermentation bed. The fermentation bed is rolled continuously so that the heat produced by fermentation is disseminated. Strains are added simultaneously so that the urine and feces are converted to organic fertilizer.

Chun’ran has a special way of establishing a sustainable production base. Researchers from universities found that adding tea leaf extracts to feed can help reduce the odor of pork, which is deemed unpleasant by some Chinese consumers. Thus, Chun’ran collaborates with researchers from universities in Zhejiang and the Ke’sheng feed company to add tea leaf extracts to feed for pigs. The tea trees are planted outside the pigpens; the practice has two advantages in terms of sustainability. First, the production waste can be used as fertilizer for those tea trees after proper disposal. Second, tea trees can absorb the smell of feces and urine excreted by the pigs to a certain extent.

#### 5.2.3. Capability in Prevention of Epidemic Diseases

Mu’yuan is good at examining vaccination, and they are strict with vaccination quality when making purchasing decisions. The domestic vaccination market is mixed with high-quality and low-quality products. Mu’yuan uses a combination of domestic and imported vaccinations in order to ensure that the pigs are well protected. Dead pig bodies are crushed completely, and after high-temperature sterilization, they are made into organic fertilizer and biodiesel.

Wen’shi established Wen’shi College, which provides free training classes to local partner farmers. In addition, Wen’shi assigns professional veterans to live near the family-owned farms in case of emergency. There is a strict sterilization system on the pig farm: Whenever people enter a farm, everything should be sterilized. In order to make farmers use vaccinations in the correct way, Wen’shi removes the drugs from farmers when pigs reach 140 days because the pigs need a period of withdrawal from the drugs.

*“Every time when people exit and enter a farm, we require them to sterilize, wash hands, and put on pasteurized clothes. The veteran personnel of Wen’shi provides us with constant guidance on how to protect the farm from being influenced by epidemic disease, and we have been disease-free for at least six months,”* said Jin Yunhao, a core family-owned commercial farmer of Wen’shi.

Long’zhu cooperative members are large-scale producers, so they have gained many skills and experience in raising pigs. Chun’ran emphasizes collaboration with universities in Zhejiang province. Based on the technological support from universities, Chun’ran formed a specialized veteran team as consultants for cooperative member farmers so that the farmers constantly receive professional assistance.

### 5.3. Supply Chain Sustainability

Through close coordination with—and education from—the core companies, farmers also achieve sustainable performance. Based on the literature review, this study adopts three dimensions to describe supply chain sustainability: The economic performance, social performance, and environmental performance of both the farmers and the company (see Table 4).

In terms of economic performance, this study uses productivity and profitability as two dimensions. Regarding productivity, Mu’yuan farmers have enhanced their capability to produce more pigs in safer ways through coordination with Mu’yuan, and Mu’yuan has succeeded in ensuring the safe production of pigs by having closer integration with its farmers. Meanwhile, Mu’yuan has stabilized its pig supply. Wen’shi farmers have substantially reduced their production costs. By constructing the production base in a more effective way, the farmers are able to raise 10% more pigs than before, and the utility costs are reduced by almost 15%. For Chun’ran, farmers are more productive because they are familiar with more good practices (i.e., farm management and treatment of pigs that died of illness) after joining the Long’zhu cooperative.

With regard to profitability, farmers from Mu’yuan benefit through coordination, are less exposed to market price risks, and their income from producing pigs was enhanced. Mu’yuan also stabilizes its profits from having good relationships with the farmers. The Wen’shi farmers have largely increased and stabilized their income in raising pigs, and it is reported that farmers never lose revenue after collaborating with Wen’shi. Chun’ran members have reduced 15% of their feed costs, and they can charge a 20% higher price premium through the tea leaf pork they produce. Thus, their profits are increased.

In terms of social performance, this study adopts two dimensions: Social welfare improvement and local community development. As a leading pig producer in China, Mu’yuan has taken great social responsibility in paying attention to environmental protection and quality enhancements. Wen’shi also addresses food safety and quality in pig production, and encourages female farmers to participate in the management and operation of family-owned commercial farms. The recycling system of production wastes innovated by Chun’ran has wide social impacts, and staff from provincial livestock bureaus in Hunan and Hubei have come to learn the wastes disposal management experience.

Mu’yuan has contributed to the development of the local community by involving local farmers in pig production and helping them to increase their income. Wen’shi has nurtured 20 family-owned commercial farms in the Huai’an area. Chun’ran has positively contributed to local development by establishing the Long’zhu cooperative, which is a national leading cooperative in China.

In terms of environmental performance, we used waste reduction and the disposal of dead pigs from illness as two dimensions. For waste reduction, all three companies have successfully found ways to reduce production wastes, and they have achieved almost zero pollution, meaning that all wastes are recycled.

Regarding the treatment of pigs that died from illness, Mu’yuan has a very advanced method of response: The dead pigs are ground down in a machine to produce organic fertilizer and biodiesel. Mu’yuan have indicated that this treatment of dead pigs achieves zero transmission of epidemic diseases. Wen’shi has determined its own method to dispose of pigs that die from illness. The CEO (Mr. Wang’ Sifeng) has reported that Wen’shi uses septic chemicals to dispose of dead pig bodies, which is environmentally sustainable. Chun’ran delivers the dead pig bodies to the collection team from the local government to have them treated.

## 6. Development of Pathways Toward Sustainable Livestock Supply Chain

Based on the analysis, it was found that there are several important steps that contribute to achieving livestock supply chain sustainability.

**Figure 1:**
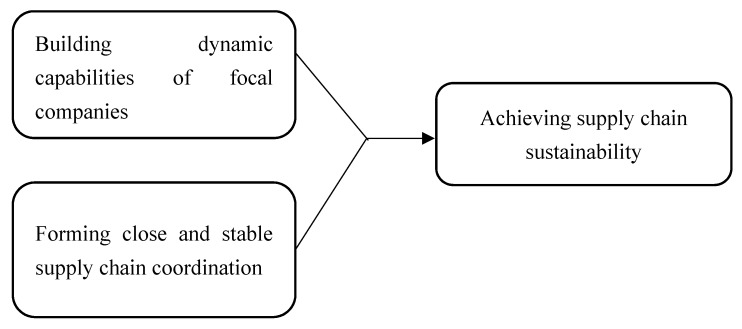
Pathway to achieve supply chain sustainability.

The first two important steps include building the dynamic capabilities of focal companies and forming close and stable supply chain coordination. In our study, the companies have strived to establish dynamic capabilities in sustainable development; they have also created a close and stable coordinative relationship with their upstream farmers. The third step is to enhance the dynamic capabilities at the supply chain level. In our study, the upstream farmers have enjoyed the learning process and gained benefits from coordinating with the focal companies. When farmers’ sustainable practices improve, the dynamic capabilities of the entire supply chain are enhanced. Finally, the entire supply chain achieves sustainable performance in improving economic interests, social welfare, and environmental protection and recycling. The path to achieving a sustainable livestock supply chain is shown in Figure 1.

## 7. Conclusions

This study reached several conclusions for the sustainable development of the livestock sector in China.

First, focal companies in the Chinese livestock supply chains are important organizations in changing the sustainable practices of stakeholders in the supply chain. Companies use their resources and capabilities to innovate and drive the sustainability of the supply chain. Some notable sustainable practices that the focal companies adopted are as follows: (i) Strictly controlling the production pollution and implementation of recycling, (ii) managing epidemics of disease according to the disease environment of the farms, and (iii) recyclable disposal of the pigs that die from illness.

Second, using supply chain coordination, focal companies transfer their good sustainable practices to other stakeholders, especially to upstream farmers with whom they collaborate. Effective coordination includes vertical integration, “focal company + family-owned commercial farms,” and “focal company + cooperatives + farmers.” The coordination serves as a learning environment for farmers to gain sustainable development capabilities, and thus the entire supply chain becomes more sustainable.

Based on these conclusions, we present some implications of this study:

First, to encourage the focal companies to enhance their sustainable practices, core companies need to tentatively follow advanced sustainable practices in the industry. Learning and innovation are also important for companies to stay alert to new sustainable production technologies. The government can provide financial and technological support to core companies to nurture their capabilities in sustainable development.

Second, core companies in the pork supply chain in China can continue to increase and innovate supply chain coordination to improve supply chain sustainability. The coordination methods examined in this study can serve as examples for core companies which do not have supply chain coordination to adopt and test. To encourage close coordination through the pork supply chain, the government may provide training on how to coordinate through supply chain to farmers so that the willingness of farmers to join farmers’ organizations increase. Core companies need to make efforts to coordinate and to constantly make this coordination efficient and effective.

## Figures and Tables

**Table 1:** Case description.

Name	Location	Year Established	Number of Interviewees
Mu’yuan	Nan’yang, Henan	1992	4
Wen’shi (Huaiyin)	Huai’an, Jiangsu	2006	6
Chun’ran	Long’you, Zhejiang	2010	5

**Table 2:** Supply chain coordination.

Name	Form of Supply Chain Coordination Mode	Main Activities
Mu’yuan	“Vertical integration”	Employ farmers to produce pigs for them on farms of Mu’yuan
Wen’shi	“Company + local family-owned commercial farms”	Sign contracts to share profits and risk with locally contracted farmers
Chun’ran	“Cooperative + farmers + company”	Purchase feed products together from Ke’sheng feed company

**Table 3:** Dynamic capabilities of focal case companies.

Name	Capability in Disposing Wastes	Capability in Establishing Sustainable Production Base	Capability in Prevention of Epidemic Diseases
Mu’yuan	Organic fertilizer and methane	Ventilation system; Humidity device; Pig house heating and insulation device	A combined use of domestic and imported vaccination; Dead pig bodies used as organic fertilizer and biodiesel
Wen’shi	Urban sewage treatment system is applied; Centralized liquid spray system; vertical fermentation tank	Leaking plate; Urinary septic tank; Fermentation bed	Free training classes to local partner farmers; Strict sterilization system
Chun’ran	Kai’qi Energy to recycle wastes into fertilizer and electricity	Tea leaf feeds for pigs; Organic fertilizer for tea trees, which absorbs the smell of feces and urine	Technological support from universities; A specialized veteran team; Withdrawal time of drugs

**Table 4:** Supply chain sustainability.

Name	Economic Performance (Productivity and Profitability)	Social Performance (Social Welfare Improvement and Local Community Development)	Environmental Performance (Wastes Reduction and Dead Pig Treatment)
Mu’yuan	Farmers: More pigs produced in safer ways; Less exposure to market price risks; Farmers’ incomes are enhanced Company: Ensure the safe production of pigs stabilize pig supply	Farmers: Gain good practice in producing high-quality pigs Company: Take great social responsibility in paying great attention to environmental protection and quality enhancing	Farmers: Collectively follow the environmental protection practices Company: The dead pigs are ground down in a machine and reproduced into organic fertilizer and biodiesel
Wen’shi	Farmers: 10% more pigs than in the same place before; Costs of utility are almost reduced by 15%; Never lose profits after contacting with Wen’shi Company: To stabilize the pig supply	Farmers: Female farmers actively participate in the management and operation of family-owned commercial farms Company: Address the food safety and quality in pig production; Pay attention to social responsibility	Farmers: Collectively follow the Wen’shi instructions Company: Use septic chemicals to dispose of dead pig bodies
Chun’ran	Farmers: More productive through knowing more good practices after joining Long’zhu; Reduced 15% of feed costs; 20% more price premium through tea-leaf pork Company: Premium pork market leader	Farmers: Know-hows in safe production Company: Long’you area is now a community that provincial livestock bureaus in Hunan and Hubei came to learn the wastes disposal management experience	Farmers: Deliver the dead pig bodies to the collection team from the local government to get them treated Company: Recycling the wastes through Kai’qi
